# Free and partially encapsulated manganese ferrite nanoparticles in multiwall carbon nanotubes

**DOI:** 10.3762/bjnano.11.170

**Published:** 2020-12-29

**Authors:** Saja Al-Khabouri, Salim Al-Harthi, Toru Maekawa, Mohamed E Elzain, Ashraf Al-Hinai, Ahmed D Al-Rawas, Abbsher M Gismelseed, Ali A Yousif, Myo Tay Zar Myint

**Affiliations:** 1 Department of Physics, Sultan Qaboos University, Muscat PC 123, Sultanate of Oman; 2 Bio-Nano Electronics Research Center, Toyo University, 2100, Kujirai, Kawagoe, Saitama 350 8585, Japan; 3 Department of Chemistry, Sultan Qaboos University, Muscat PC 123, Sultanate of Oman

**Keywords:** carbon nanotubes, charge transfer, manganese ferrite, metallic nanoparticles, partial encapsulation, stress, surface

## Abstract

Free and partially encapsulated manganese ferrite (MnFe_2_O_4_) nanoparticles are synthesized and characterized regarding structure, surface, and electronic and magnetic properties. The preparation method of partially encapsulated manganese ferrite enables the formation of a hybrid nanoparticle/tube system, which exhibits properties of manganese ferrite nanoparticles inside and attached to the external surface of the tubes. The effect of having manganese ferrite nanoparticles inside the tubes is observed as a shift in the X-ray diffraction peaks and as an increase in stress, hyperfine field, and coercivity when compared to free manganese ferrite nanoparticles. On the other hand, a strong charge transfer from the multiwall carbon nanotubes is attributed to the attachment of manganese ferrite nanoparticles outside the tubes, which is detected by a significant decrease in the σ band emission of the ultraviolet photoemission spectroscopy signal. This is followed by an increase in the density of states at the Fermi level of the attached manganese ferrite nanoparticles in comparison to free manganese ferrite nanoparticles, which leads to an enhancement of the metallic properties.

## Introduction

Since the discovery of carbon nanotubes (CNTs), researchers have been interested in functionalizing, inserting, and encapsulating materials inside their inner cavities. Processes related to functionalizing and inserting nanomaterials (i.e., directly implanting as-synthesized nanomaterials into CNTs) have been widely reported in the literature are and generally compared to encapsulation processes (i.e., in situ syntheses of nanomaterials in CNT cavities). The latter suffers from a lack of tube-filling capacity along with unknown interactions between the inner parts of the tube and the hosted materials. Despite these drawbacks, it has been suggested that encapsulating materials into the hollow regions of carbon nanotubes can result in a significant change of the properties of the inserted materials [1–3]. Manganese ferrite nanoparticles can be used in biomedical diagnostics and have good properties as contrast agents [4]. In addition, encapsulating magnetic nanoparticles inside carbon nanotubes enables the handling of the tubes via magnetic forces, thereby avoiding the alteration of their electronic or mechanical properties when using them in nanoelectronics [5]. Moreover, carbon nanotubes filled with magnetic materials have the potential to be used for the transport of drugs to specific locations in the body as well as in medical diagnosis without the need for surgical interference [6]. Pal et al. synthesized Fe_3_O_4_ encapsulated in carbon nanostraws and reported an enhancement in the magnetic properties, which were attributed to an increase in the dipolar interparticle interactions due to the close packing of nanoparticles within the tubes [7]. There are several potential applications that use metal–metal oxide/CNTs hybrid systems. Carbon nanostructures decorated with titania and silica are used for the removal of harmful pollutants [8], carbon nanotubes decorated with molybdenum disulfide are used in microbial fuel cells [9] and photoelectric detectors [10], supercapacitor electrodes use binary metal oxide/multiwall carbon nanotubes (MWCNTs) [11], among other numerous recently developed applications [12].

Although the independent benefits of the functionalization, insertion, and encapsulation of nanomaterials have been identified, to the best of our knowledge, there are no studies exploring the combined advantages of having nanomaterials simultaneously located inside and outside (attached to the external surface) of the tubes. This study is the first attempt to synthesize and investigate structural, surface, electronic, and magnetic properties of such a hybrid manganese ferrite nanoparticles/MWCNT system, called partially encapsulated manganese ferrite nanoparticles, and compare their properties with free manganese ferrite nanoparticles of a similar size. The hybrid synthesis method (partially encapsulated manganese ferrite nanoparticles/MWCNT system) enables the simultaneous formation of nanoparticles inside the tube cavities and attached to the external surface of the tubes. Therefore, it allows for the investigation of possible effects associated with the location of these nanomaterials.

## Experimental

### Synthesis

The free manganese ferrite nanoparticles were prepared by adding, dropwise, 100 mL of a solution containing 0.10 M of Fe^3+^ and 0.05 M of Mn^2+^, to 100 mL of a boiling solution containing 0.45 M of sodium hydroxide, under constant stirring. After the addition, the mixture was covered and aged in an oven at 373 K for two days. The reaction vessel at the end of the aging period was taken out of the oven and cooled down to room temperature. The mixture was centrifuged, washed three times with distilled water, and dried in an oven at 373 K for 24 h.

The partially encapsulated manganese ferrite nanoparticles were prepared in the presence of MWCNTs, purchased from the Chinese Academy of Sciences (CAS), with an inner diameter of 5–10 nm and an outer diameter of 20–30 nm. A round-bottom flask containing MWCNTs (0.5 g), manganese nitrate (Mn(NO_3_)_2_·6H_2_O, 2.0 g) and iron nitrate (Fe(NO_3_)_3_·9H_2_O, 5.6 g) dissolved in 18.0 mL of nitric acid (69%) was heated to reflux for 4.5–5 h. The nitric acid solution was decanted and the black sludge was filtered through a glass fiber filter paper. A vacuum filter flask was used and the sample was kept under filtration for 6 h to remove the remaining nitric acid along with existing dissolved materials. The sample was oven-dried at 373 K and calcined in a stream of N_2_ at 673 K, for 4 h, for the conversion of nitrates into ferrites.

### Structural, surface, electronic, magnetic, and Mössbauer characterization

The X-ray diffraction patterns were acquired using Cu Kα radiation (λ = 1.5404 Å) at a Philips PW1820 diffractometer. The instrument was calibrated using a silicon disc. Free manganese ferrite nanoparticles were characterized using high-resolution transmission electron microscopy (HRTEM) on a JEOL JEM-2100F microscope working at 200 kV. Partially encapsulated manganese ferrite nanoparticles were characterized using HRTEM and energy-dispersive X-ray spectroscopy (EDS); the elemental mapping was performed on a JEOL JEM-ARM200F. Scanning transmission electron microscopy (STEM) bright-field and dark-field images were acquired at 80 kV. MnFe_2_O_4_ nanoparticles and MnFe_2_O_4_/MWCNTs samples were dispersed in absolute ethanol and sonicated for 15 min to obtain a homogenous dispersion. Then, the supernatant was micropipetted onto a TEM grid and dried at room temperature (approx. 300 K) overnight.

X-ray photoelectron spectroscopy (XPS, Omicron Nanotechnology) was employed with a monochromatic Al Kα radiation (*h*ν = 1486.6 eV), with a source voltage of 15 kV and an emission current of 20 mA. Scans were carried out at a base pressure of 2 × 10^−8^ Pa. A wide scan was recorded at a constant analyzer transmission energy of 50 eV, while the individual elemental peaks were recorded at an analyzer pass energy of 20 eV. All XPS measurements were carried out at room temperature and no heating was performed prior to the XPS measurement. The obtained XPS spectra were deconvoluted using the CasaXPS program (Casa Software Ltd., UK), in which the background was simulated using the Shirley function and the peaks were fitted using the Gaussian–Lorentzian function. The recorded spectra were corrected using the binding energy of aliphatic carbon at 285 eV and the accuracy of the measured binding energy values was estimated to be equal to ±0.1 eV. Due to the charging effect of the oxides, electron flooding was carried out for charge compensation. A helium lamp with 21.2 eV (He I) excitation energy was used for ultraviolet photoemission spectroscopy (UPS, Omicron Nanotechnology). A −5 V sample bias was applied during work function measurements to separate the sample and analyzer spectral cutoffs.

Hysteresis loops at 300, 77, and 4 K and zero-field cooling curves were recorded using a superconducting quantum interference device (SQUID). Mössbauer spectra were obtained for the powdered samples at 300 and at 77 K, using a Mössbauer spectrometer in constant-acceleration mode with 50 mC ^57^Co in a Rh source.

## Results and Discussion

### Free MnFe_2_O_4_ nanoparticles

**Figure 1:**
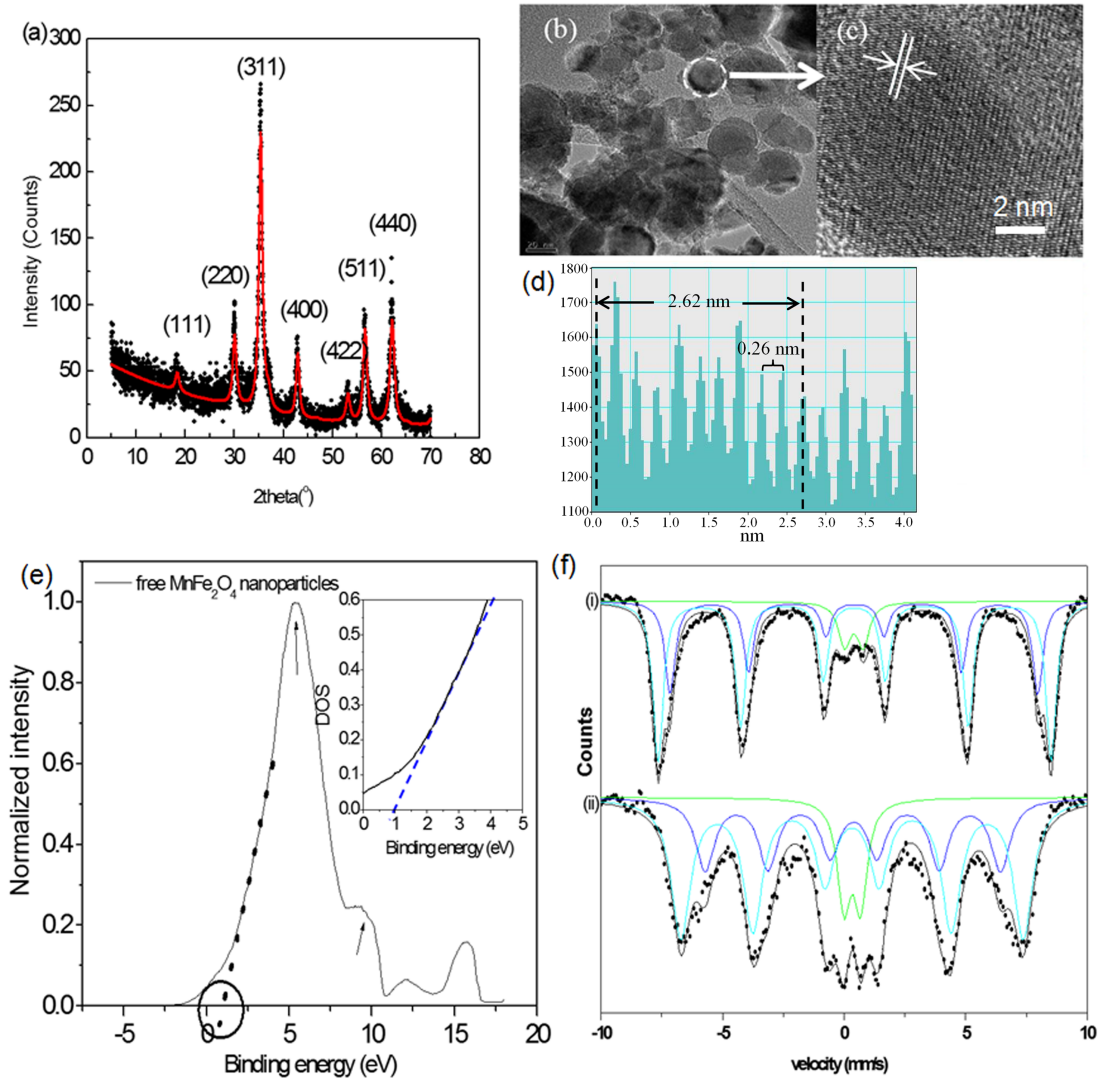
(a) Rietveld refined pattern of XRD for free MnFe_2_O_4_ nanoparticles. (b) TEM image of free MnFe_2_O_4_ nanoparticles. (c) HRTEM image of free MnFe_2_O_4_ nanoparticles showing a lattice spacing of the (311) planes. (d) Line profile shown in (c). (e) UPS spectrum of free MnFe_2_O_4_ nanoparticles. The circle indicates the estimated valence band maximum value. Inset: DOS at the Fermi level. (f) Mössbauer spectra of free MnFe_2_O_4_ nanoparticles at (i) 77 K and at (ii) 300 K.

The XRD patterns were analyzed using the MAUD (material analysis using diffraction) fitting software based on the Rietveld method and on Fourier analysis. The analysis was carried out to obtain the crystallite size and lattice parameters. For that, a pseudo-Voigt peak-shape profile was used in which an iterative least-square procedure was adopted through the minimization of the residual parameters [13]. X-ray diffraction data (Figure 1a) confirmed the cubic spinel structure of free MnFe_2_O_4_ nanoparticles with a crystallite size of 12 ± 1 nm. The lattice parameter was found to be 0.8488 nm, which is in good agreement with the reported value of 0.8499 nm for bulk MnFe_2_O_4_ [14]. A careful analysis of the TEM images indicated that MnFe_2_O_4_ nanoparticles have sizes ranging from 5 to 24 nm, with an estimated average size of 10–19 nm (Figure 1b). The particle size distribution is shown in Supporting Information File 1, Figure S2. In addition, a HRTEM image (Figure 1c) indicates a lattice spacing of 0.26 nm for the (311) plane of the MnFe_2_O_4_ nanoparticles [15], as confirmed by the line profile shown in Figure 1d.

In order to investigate the electronic properties of free MnFe_2_O_4_ nanoparticles, ultraviolet photoelectron spectroscopy was employed. Even though theoretical studies have been reported for MnFe_2_O_4_ [16], to the best of our knowledge, these are the first UPS measurements carried out for this system. Figure 1e provides the structure of the valence band of free MnFe_2_O_4_ nanoparticles. Experimental results indicate that the density of states (DOS) at the Fermi level, *E*_F_ (i.e., at a zero binding energy in the UPS spectrum) is consistent with that of half-metallic behavior (Figure 1e, inset). Ideally, stoichiometric MnFe_2_O_4_ is predicted to be a half metal with a low carrier density [17]. A valence band maximum of 0.85 eV, derived from the spectrum, is in line with its predicted half-metallic behavior. However, it is to be noted that, experimentally, bulk MnFe_2_O_4_ is known to have semiconductor properties [18]. The spectrum shown in Figure 1e contains a main peak at approx. 5.4 eV and weaker peaks at approx. 9.7 eV and 12.1 eV. By comparing the spectrum with the band structure calculations, the first peak (indicated by an arrow in the spectrum) can be attributed to the electrons emitted from the Mn 3d states [19] and the second peak (also indicated by an arrow in the spectrum) is due to the localization of the Fe 3d electrons at an energy level of 8 eV, which is far away from the Fermi level [16].

**Figure 2:**
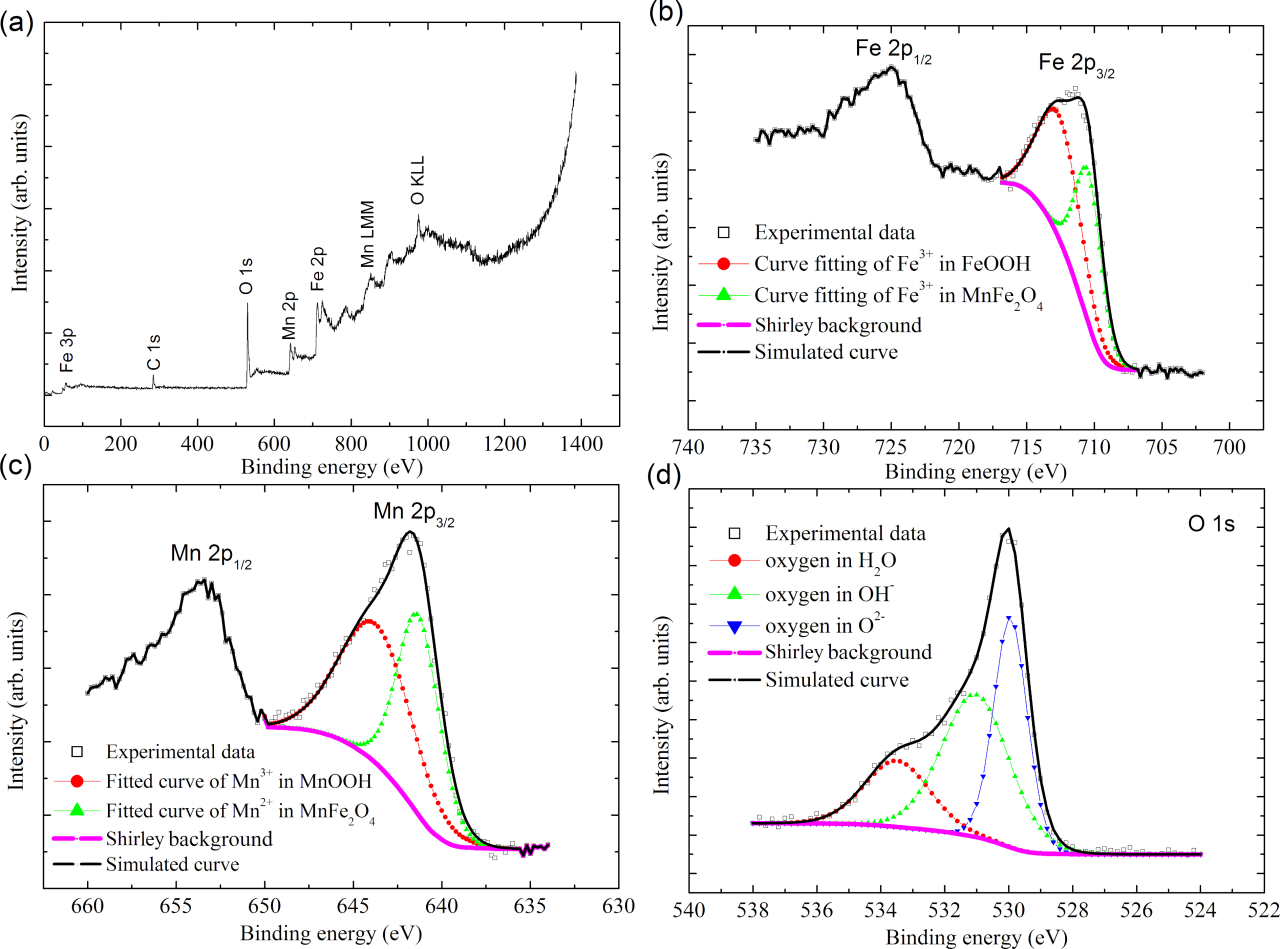
Core level XPS spectra of free MnFe_2_O_4_ nanoparticles: (a) XPS survey scan of free MnFe_2_O_4_ nanoparticles. (b) Fe 2p, (c) Mn 2p, and (d) O 1s.

To fit the measured Mössbauer spectra of free MnFe_2_O_4_ nanoparticles, two different subspectra associated with tetrahedral (A) and octahedral (B) sublattices, with close but different hyperfine fields, were used in addition to a superparamagnetic doublet (Figure 1f). Isomer shift values reflect the presence of high-spin Fe^3+^ ions [14], whereas Fe^2+^ is undetectable, as shown in Table 1. These results indicate that the divalent cations in free MnFe_2_O_4_ nanoparticles are Mn^2+^, which supports the XPS data in Figure 2.

**Table 1:** Mössbauer parameters of free manganese ferrite nanoparticles. δ (±0.02) is the isomer shift, Δ*E*_Q_ (±0.02) is the quadruple splitting, Г (±0.01) is the line width, *H*_hf_ (±0.2) is the hyperfine field, area (±1) is the relative spectra area, and D indicates the doublet component of the spectra.

*T* (K)	δ (mm/s)			Δ*E*_Q_ (mm/s)			Г (mm/s)		*H*_hf_ (T)		Area (%)		
					
	A	B	D	A	B	D	sextets	D	A	B	A	B	D

77	0.43	0.43	0.40	−0.02	0.00	0.76	0.50	0.60	46.9	50.1	32	59	8
300	0.37	0.33	0.34	−0.02	0.00	0.69	0.47	0.64	37.7	43.8	32	57	11

The Mössbauer spectrum in Figure 1f shows sextets, which are attributed to the field arising from larger particles superimposed on a relatively small superparamagnetic doublet due to the presence of very small crystallites [20]. The values of hyperfine fields are smaller than those obtained for bulk MnFe_2_O_4_ [21]. The reduction in the hyperfine field values follows the change in degree of inversion of the nanoparticles, in which Fe^3+^ occupation is 32% (A) in comparison to 20% of the bulk occupation (A) [21–22]. Quadrupole splitting and line width parameters are consistent with those reported for MnFe_2_O_4_ nanoparticles [14].

The elements Mn, Fe, and O can be clearly identified from the XPS survey spectrum (Figure 2a). The core level spectrum of Fe 2p is presented in Figure 2b. On the deconvolution of Fe 2p_3/2_, peaks are observed at 710.4 and at 712.6 eV. The first peak can be attributed to Fe^3+^ in free MnFe_2_O_4_ nanoparticles, whereas the second peak corresponds to Fe^3+^ in FeOOH [23], resulting from the adapted preparation method. Similarly, on the deconvolution of Mn 2p_3/2_ the peak at 641.3 eV corresponds to the binding energy of Mn^3+^ in manganite (MnOOH) (Figure 2c). The deconvoluted Mn 2p_3/2_ peak at 643.7 eV can be assigned to Mn^2+^, in accordance with the binding energy of Mn^2+^ in MnO, which is generally found at 641.5 eV. Therefore, it can be concluded that manganese exists as Mn^2+^ and iron exists as Fe^3+^ in free MnFe_2_O_4_ nanoparticles.

It is observed from the deconvolution of the O 1s peak (Figure 2d) that oxygen is present in three environments. The peak with the lower binding energy at 529.9 eV can be associated with oxygen in the crystal lattice O^2−^ [24]. The deconvoluted peak at 531.0 eV can be assigned to OH^−^ [25], arising from the incorporated preparation methodology, and the peak at 533.5 eV can be assigned to O in H_2_O [26].

**Figure 3:**
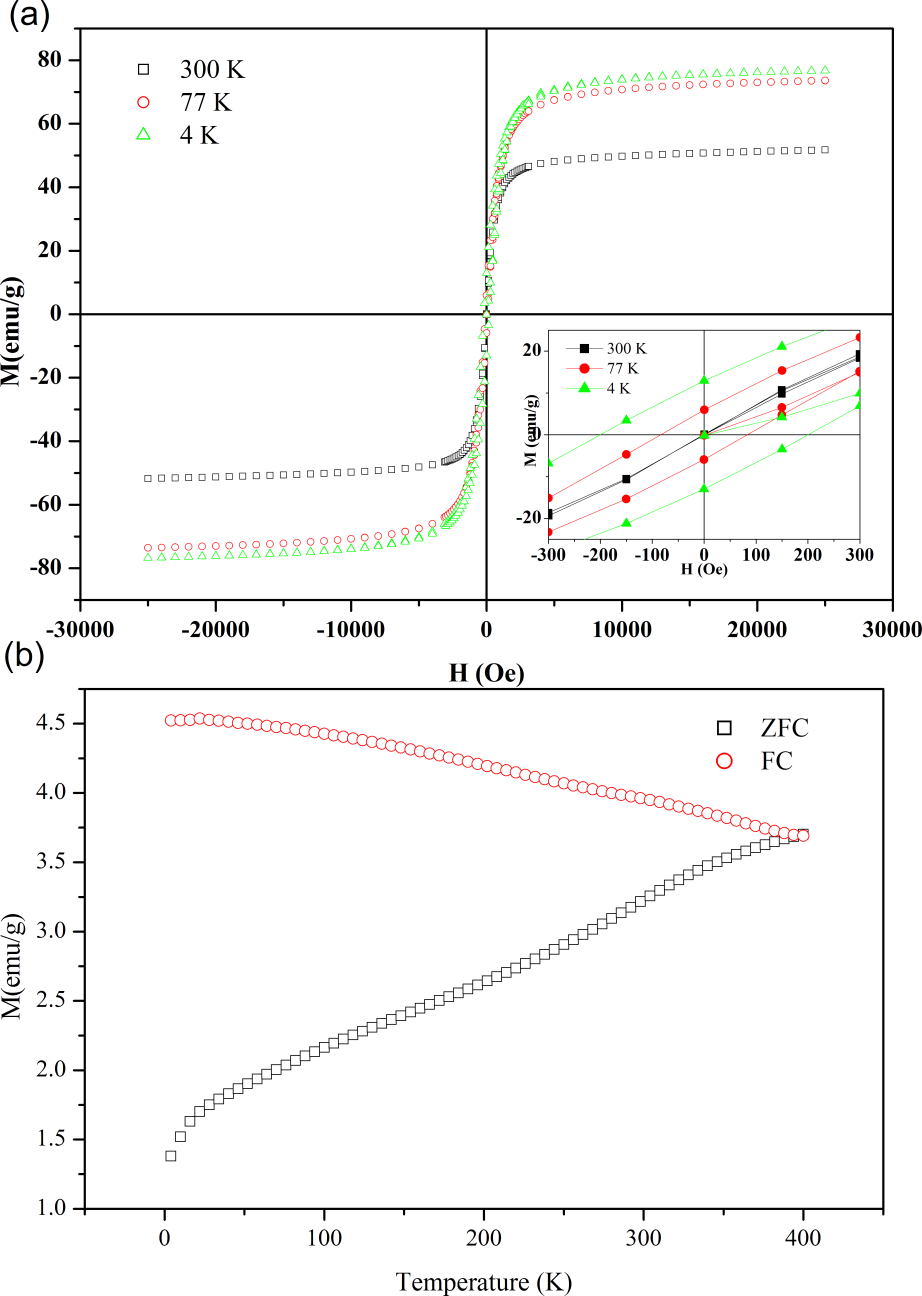
(a) *M*–*H* loops of free MnFe_2_O_4_ nanoparticles at 300, 77, and 4 K. The inset shows as enlarged view of *M*–*H* loops. (b) Magnetization measurements under ZFC and FC conditions in an applied magnetic field of 50 Oe.

At room temperature, free MnFe_2_O_4_ nanoparticles display both ferrimagnetic and superparamagnetic behavior (Figure 3a). The saturation magnetization at room temperature, with an observed value of 52 emu/g, is lower than the reported bulk value of 80 emu/g [20]. This saturation magnetization value is reported for particles with 11 nm in size [27], which is approximately the average size of our nanoparticles (10–19 nm), as discussed in the analysis of Figure 1b. The decrease in saturation magnetization of the nanoparticles, compared to the bulk, is attributed to the effect of a weak magnetic layer generated by FeOOH and MnOOH, which exists on the surface of the nanoparticles [27]. The superparamagnetic behavior observed at 300 K arises from the small sizes of the particles in view of the known 8 nm superparamagnetic diameter of MnFe_2_O_4_ [27]. This observation is supported by the Mössbauer data, where, at 300 K, a doublet of 11% adsorption area originating from small particles, in addition to the sextets, is detected (Figure 1f).

Hysteresis loops at 77 and 4 K display an increase in magnetization, compared to the hysteresis loop at 300 K, as a result of the decrease in thermal energy as the temperature decreases [20]. As the temperature decreases, the coercivity increases (Figure 3a, inset). The coercivity at 77 K is 83.5 Oe and at 4 K is approx. 200.6 Oe, which is the value found for capped MnFe_2_O_4_ nanoparticles with sizes of 4.6–9.9 nm [28].

The blocking temperature, *T*_B_, is obtained from the maximum of the zero-field cooling curve (ZFC) (Figure 3b). The magnetization initially increases with the temperature until it reaches a maximum value at approx. 400 K, a point at which both ZFC and the field cooling (FC) curve converge. This *T*_B_ value is expected for nanoparticles with a large size of approx. 16 nm [28]. Above the blocking temperature, free MnFe_2_O_4_ nanoparticles are characterized by the superparamagnetic behavior.

### Partially encapsulated manganese ferrite nanoparticles inside multiwall carbon nanotubes

**Figure 4:**
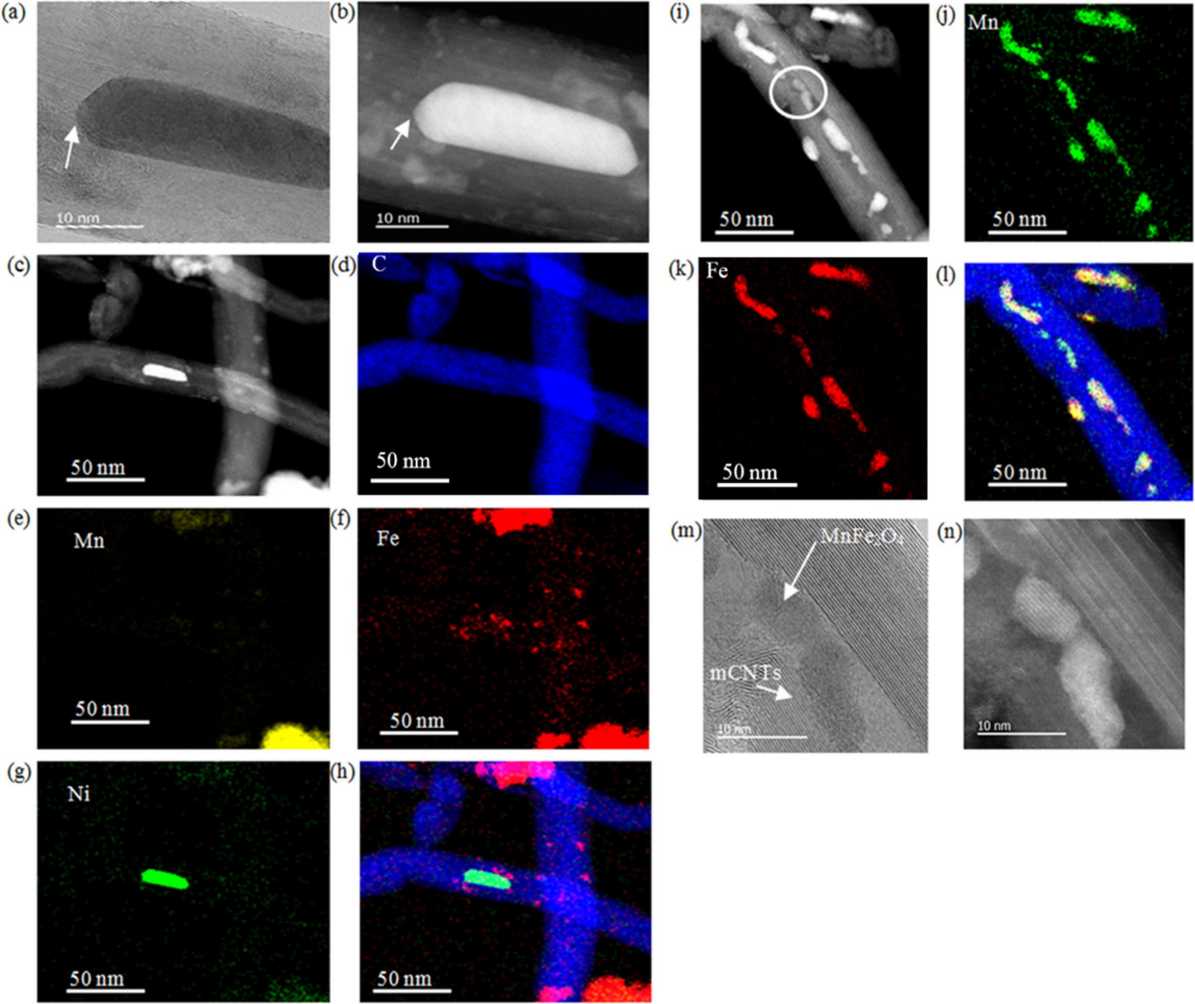
(a) Dark-field STEM image. (b) Bright-field STEM image. (c) Annular dark-field image. EDS mapping of (d) carbon, (e) manganese, (f) iron, (g) nickel (the source of Ni is the original MWCNTs used as catalyst), and (h) overlays of MnFe_2_O_4_/MWCNTs. The arrows in (a) and (b) are pointing at MWCNTs layers, which are coating the partially encapsulated nanoparticles. (i–l) The majority of the MnFe_2_O_4_ nanoparticles are in the inner part of the tubes. (m,n) HRTEM images at the location identified by a circle in (i).

The formation of MnFe_2_O_4_ nanoparticles inside the inner cavity of MWCNTs and outside of MWCNTs is confirmed by chemical mapping using EDS–STEM (Figure 4). The particles have an elongated shape, expanding longitudinally in the available hollow space. In the transverse direction, the particle is restricted by the walls of the MWCNTs. The residual Ni, left in the MWCNTs due to the adapted preparation method, are attracted to the particles inside the MWCNTs. The inner layers of MWCNTs tend to coat the ends of the particles, leading to damage (marked by arrows in Figure 4a and Figure 4b) at the inner walls of the tubes. As observed, MnFe_2_O_4_ nanoparticles can attach themselves to the outer surface of the tubes (Figure 4e–h). The majority of the MnFe_2_O_4_ particles occupy the inner hollow of the tubes and the percentage of this occupation increases as the inner diameter of the tubes gets larger (Figure 4i–l). The nanoparticles are distributed within the tube structure and the location of the formed nanoparticles depends on the location of the nitrates during the formation of partially encapsulated nanoparticles. Figure 4m and Figure 4n show the lattice planes of the manganese ferrite nanoparticles inside the tubes.

**Figure 5:**
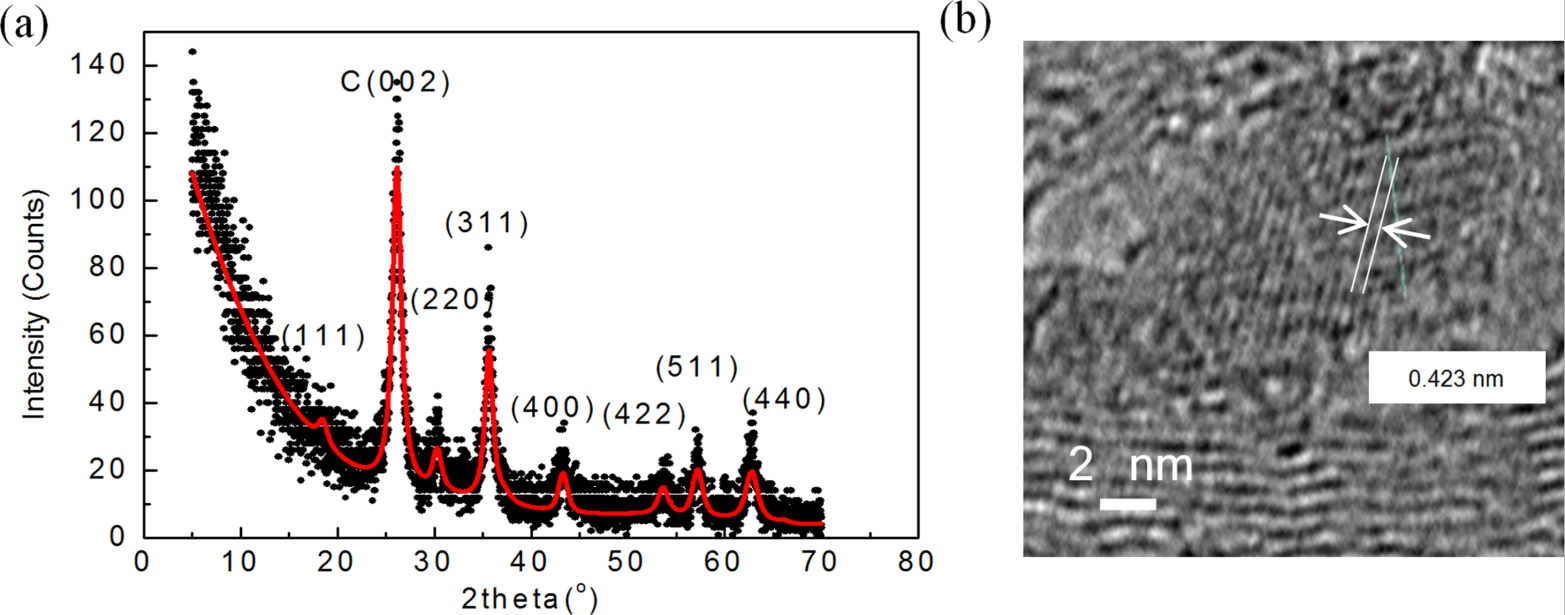
(a) Rietveld refinement of the XRD pattern of MnFe_2_O_4_/MWCNTs. (b) HRTEM image of the (111) plane showing the interfringe distance measurement.

All measurements were carried out on the sample shown in Figure 4i–l and confirmed by a shift of the XRD peaks of MnFe_2_O_4_. The X-ray diffraction pattern was refined using Rietveld refinement techniques (Figure 5a). There is a shift in the X-ray diffraction peaks of partially encapsulated manganese ferrite nanoparticles in comparison to the X-ray diffraction peaks of free manganese ferrite nanoparticles [29]. Moreover, as an example, the largest-intensity peak of partially encapsulated manganese ferrite (311) is observed at θ = 17.51°, whereas, for the free MnFe_2_O_4_ nanoparticles, it is observed at θ = 17.70°. From the refinement, the estimated crystallite size is 10 ± 1 nm and lattice parameter is 0.83992 nm. The lattice parameter is 1% smaller than the lattice parameter of the reference pattern (0.84983 nm). The HRTEM image (Figure 5b), representing the manganese ferrite inside the tube, shows lattice fringes with a measured interfringe distance of 0.42 ± 0.01 nm, which is smaller than the reported interfringe distance of 0.49 nm for the (111) planes [30].

In addition, based on the measured interfringe distance of the (111) plane, the lattice parameter calculated using the Bragg equation is 0.73 nm, which is significantly lower than the value reported in the literature. Even though images in Figure 4 clearly show the formation of manganese ferrite nanoparticles inside and outside the tubes, the shifts in the positions of the X-ray diffraction peaks, along with a decrease in the interplanar lattice spacing, are attributed to the particles located in the inner cavities of the tubes.

**Figure 6:**
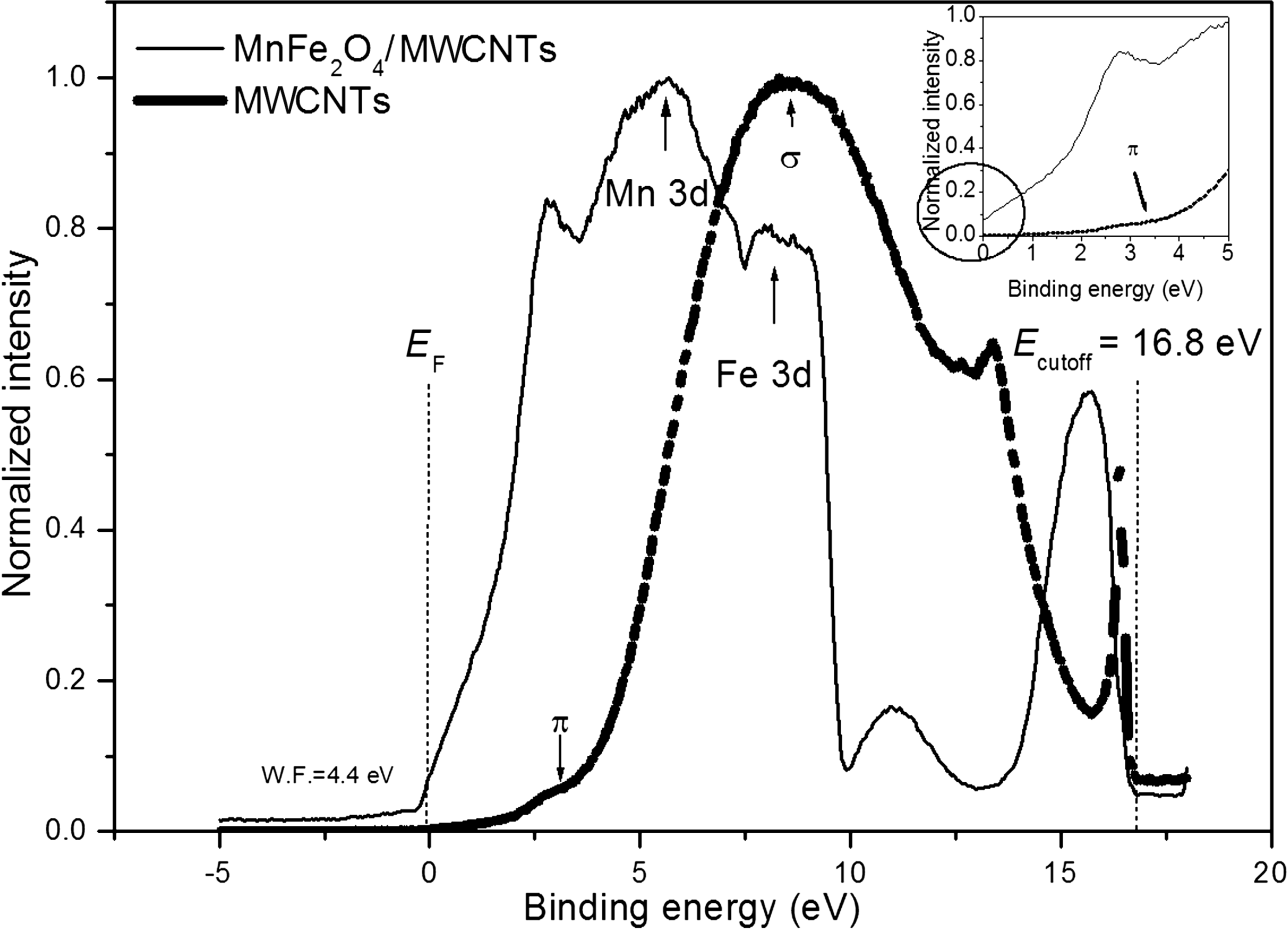
UPS spectrum of MnFe_2_O_4_/MWCNTs and pristine MWCNTs. Inset: magnified spectra near the Fermi level. The circle indicates DOS at the Fermi level.

Multiwall carbon nanotubes range from metallic to semiconducting structures [31–32]. The valence edge of the CNTs correspond to the work function [33]. The well-known features of three-fold coordination of C atoms are the deep-lying σ band, corresponding to a strong in-plane bonding located at 8.1 eV, and delocalized π bands, representing the weak bonding perpendicular to the graphene plane positioned at 3.1 eV (sp^2^-hybridized carbon network) [34]. These features can be clearly seen in Figure 6. A work function identification of MnFe_2_O_4_/MWCNTs is needed to better understand the electronic structure and the interaction between MWCNTs and MnFe_2_O_4_ at the interface. Here, we estimated the MnFe_2_O_4_/MWCNTs work function from the difference in the photon energy of 21.2 eV (He I) and from the energy difference between the secondary cutoff energy (*E*_cutoff_) and the Fermi edge (*E*_F_), which is found to be 4.4 eV. This work function value is slightly lower than that of graphite and it is explained by the destabilization of π electrons due to the curvature of the graphene sheets [35].

As observed from Figure 1e and Figure 6, there are large spectral differences between free MnFe_2_O_4_, MnFe_2_O_4_/MWCNTs, and pristine MWCNTs. Considering that the probing depth of UPS is only at the nanometer scale and the diameter values of MWCNTs are quite large (20–30 nm), it can be concluded that all features observed in Figure 6 are associated with MnFe_2_O_4_ attached to the external surface of the MWCNTs. MnFe_2_O_4_ particles inside the tubes did not contribute to the features observed. Although it is anticipated that encapsulation will cause some changes in the electronic structure in the vicinity of the particles inside the tubes, if all MnFe_2_O_4_ particles are encapsulated, the outer layers of MWCNTs will be unaffected. As a result, it would be expected that the UPS spectrum of coated MWCNTs is similar to that of pristine MWCNTs. This explanation is consistent with STEM images and EDS mappings presented in Figure 4, which show MnFe_2_O_4_ particles inside the tubes and attached to the external surfaces of the MWCNTs.

The first and the second peak in the UPS spectrum of MnFe_2_O_4_/MWCNTs (Figure 6) correspond to the convolution of the π band of MWCNTs and the Mn 3d electrons emitted from MnFe_2_O_4_, respectively. In addition, the formation of MnFe_2_O_4_/MWCNTs causes an enhancement in the DOS at the Fermi level, as indicated in the inset of Figure 6 [36]. Reports in the literature attributed the change in intensity of the emitted electrons from the π band to a strong charge transfer between MWCNTs and the functionalized materials [37]. In our case, the π band signal in MnFe_2_O_4_/MWCNTs is obscured by the presence of the Mn 3d signal, which makes it difficult to draw any conclusion related to the charge transfer. However, the UPS peak at 8 eV in the spectrum of MnFe_2_O_4_/MWCNT is composed of electrons emitted from the σ band of MWCNTs and the Fe 3d states of MnFe_2_O_4_. This peak has a lower intensity in comparison to the σ peak of the pristine MWCNTs, but has a higher intensity in comparison to the Fe 3d state in free MnFe_2_O_4_ (Figure 1e). The decrease of the σ signal and the increase of the intensity values of Fe 3d imply the possibility of a charge transfer from the MWCNTs to the MnFe_2_O_4_ attached outside of the tubes. Yu et al. reported that the charge transfer phenomenon can also be due to the interaction between the CNT walls and the encapsulated nanoparticles [38].

**Figure 7:**
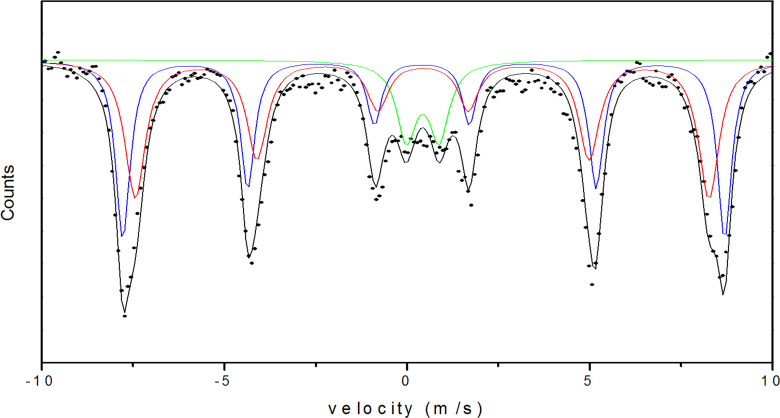
Liquid-nitrogen Mössbauer spectrum of MnFe_2_O_4_/MWCNTs.

The liquid-nitrogen Mössbauer spectrum (Figure 7) displays a superposition of two magnetic components and a superparamagnetic component. The Mössbauer parameters are presented in Table 2. The area of the doublet (superparamagnetic component) shows that 11.9% of the magnetic moments fluctuate due to the presence of very small particles [39]. The values of the isomer shifts reflect the preservation of Fe^3+^. The magnetic sextets correspond to the Fe atoms located at the tetrahedral and octahedral sites in the spinel structure of MnFe_2_O_4_. These results confirm the ferrimagnetic behavior of the as-prepared MnFe_2_O_4_. The distribution of Fe^3+^ in the sites is found from the relative area covered by the sextets. The occupation of Fe^3+^ in the tetrahedral site is 45.9% compared to the 20% occupation in bulk MnFe_2_O_4_. In addition, the hyperfine fields at the tetrahedral and octahedral sites are lower than those of the bulk [21]. The large value used for the line width is due to the large size distribution and/or to surface effects.

**Table 2:** Mössbauer parameters of MnFe_2_O_4_/MWCNTs. δ (±0.02) is the isomer shift, Δ*E*_Q_ (±0.02) is the quadruple splitting, Г (±0.01) is the line width, *H*_hf_ (±0.2) is the hyperfine field, area (±1) is the relative spectra area, and D indicates the doublet component of the spectra.

*T* (K)	δ (mm/s)			Δ*E*_Q_ (mm/s)			Г (mm/s)		*H*_hf_ (T)		Area (%)		
					
	A	B	D	A	B	D	A	B	A	B	A	B	D

77	0.42	0.43	0.43	−0.02	0.03	0.89	0.48	0.66	48.7	51.1	46	42	12

Although free and partially encapsulated manganese ferrite nanoparticles are prepared using two different methodologies, when comparing the hyperfine field of MnFe_2_O_4_ nanoparticles and MnFe_2_O_4_/MWCNTs at 77 K, there is a 4% increase observed in the hyperfine field of the partially encapsulated MnFe_2_O_4_ at the tetrahedral site and a 2% increase at the octahedral site. The occupation of Fe^3+^ at the tetrahedral site increases from 32% to 46%. This may be due to a higher calcination temperature used during the preparation of the partially encapsulated MnFe_2_O_4_ sample [20]. The origin of the magnetic hyperfine field in MnFe_2_O_4_ is the magnetic moment of the unpaired 3d electrons coupled by a super-exchange interaction via the oxygen ions that separate them. The Fe^3+^_A_–Fe^3+^_B_ exchange interaction is two times stronger than the Mn^2+^_A_–Fe^3+^_B_ exchange interaction [40]. Therefore, the increase in the number of Fe^3+^ ions occupying the tetrahedral site could be the cause of the increase observed in the hyperfine field. However, MWCNTs straining the partially encapsulated MnFe_2_O_4_ nanoparticles play an important role here. The effect of the strain in MWCNTs reflects in a 1% reduction in the lattice parameter of partially encapsulated MnFe_2_O_4_ as compared to that of free MnFe_2_O_4_. Hence, the interplanar distance values decrease and, in turn, the distance values between the magnetic ions decrease. Consequently, the strength of the interaction between the magnetic ions increases, leading to an increase of the hyperfine field at the nucleus of strained Fe^3+^ cations [41].

**Figure 8:**
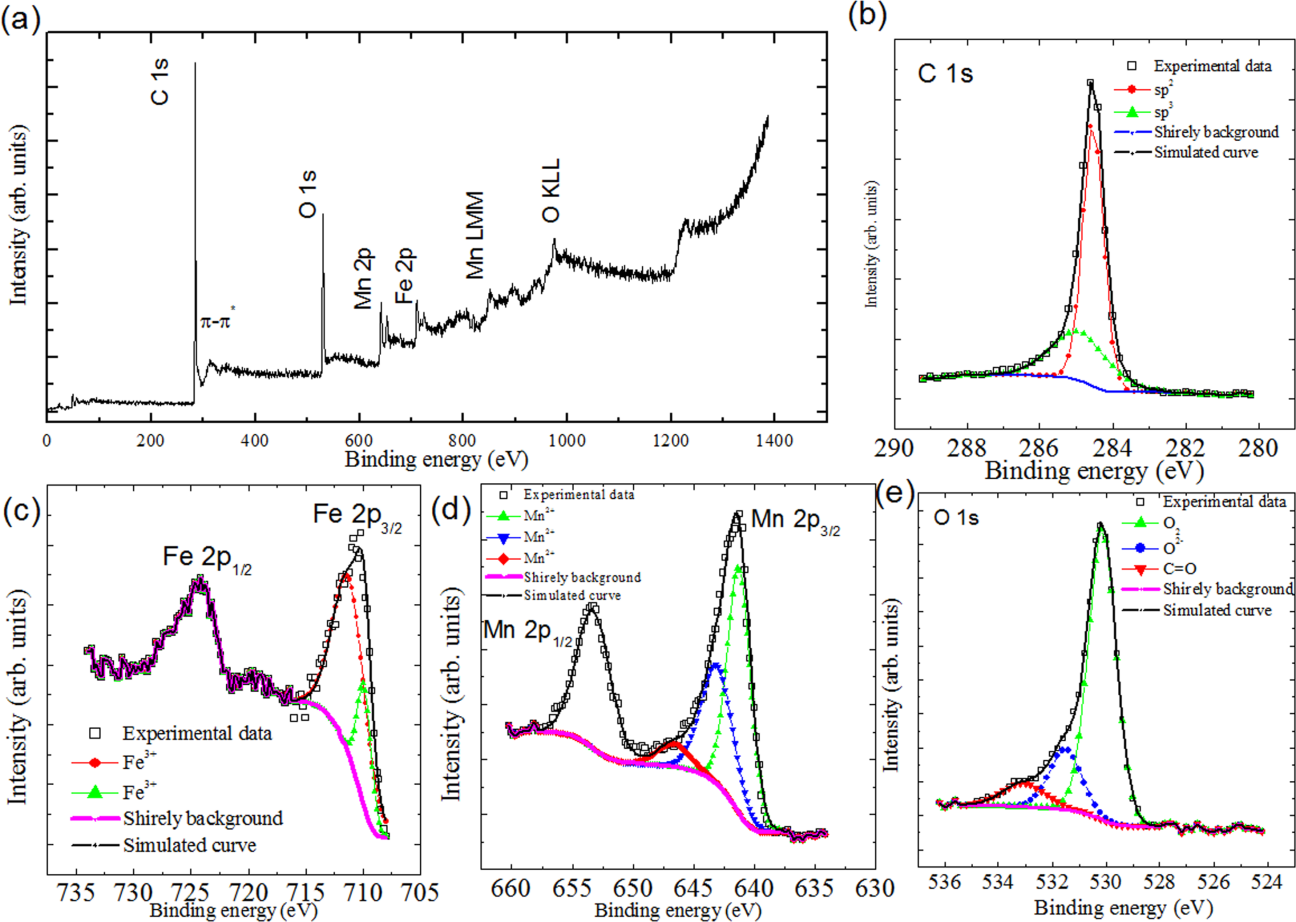
(a) Survey scan of MnFe_2_O_4_/MWCNTs. Core level XPS spectra of MnFe_2_O_4_/MWCNTs: (b) C 1s, (c) Fe 2p, (d) Mn 2p, and (e) O 1s.

Due to the nanometer scale of the effective sampling depth of XPS (approx. 10 nm), it is anticipated that all the core level spectra shown in Figure 8 are from the MnFe_2_O_4_ particles attached to external surface of the tubes rather than from those in the inner cavities of the tubes. The XPS survey scan of MnFe_2_O_4_/MWCNTs (Figure 8a) shows photoelectron lines of C, O, Mn, and Fe. The highest-intensity peak is noticed for carbon, which corresponds to the carbon content of MWCNTs. The deconvolution of the core level energy peak of C 1s identifies two components at 284.5 and 285.5 eV, respectively. The first peak is ascribed to sp^2^-hybridized and the second peak is mainly from the sp^3^ carbon [42] (Figure 8b). The evaluation of the sp^2^/sp^3^ ratio shows that there is a 1% higher transformation of sp^2^-hybridized to sp^3^-hybridized carbon in MnFe_2_O_4_/MWCNTs compared to the as-prepared samples. Thus, it is assumed that there is negligible damage to the tubes as a result of an external attachment of MnFe_2_O_4_.

The Fe 2p_3/2_ core level emission peak is detected at 710.4 eV, with simulated binding energy values of 710.3 and 712.0 eV (Figure 8c). The satellite peak is observed at 8.0 eV above the main peak of Fe 2p_3/2_, a characteristic reported for Fe^3+^ [43], whereas the Fe^2+^ 2p_3/2_ peak is reported to be associated with a satellite peak at 6.0 eV above the main peak [24]. Multiple peaks at binding energies of 710.3 and 712.2 eV are reported for Fe^3+^ 2p_3/2_ components [23]. Therefore, the presence of only Fe^3+^ could be justified. The Mn 2p_3/2_ core level emission peak is localized at 641.2 eV, with components at 641.0, 642.4, and 644.6 eV. The observed binding energies are reported for the Mn^2+^ 2p_3/2_ peak [24] and multiplet constituents [23] (Figure 8d). Hence, it could be inferred that manganese is present in the Mn^2+^ state. Three photoemission peaks, illustrated in the simulation of the O 1s peak in Figure 8e, can be assigned to three distinct oxygen species. The main peak at 530.1 eV corresponds to the crystal lattice oxygen O^2−^. The peak at 531.5 eV can be attributed to adsorbed molecular oxygen [24] and the third peak at 533.1 eV is common for C=O [44].

**Figure 9:**
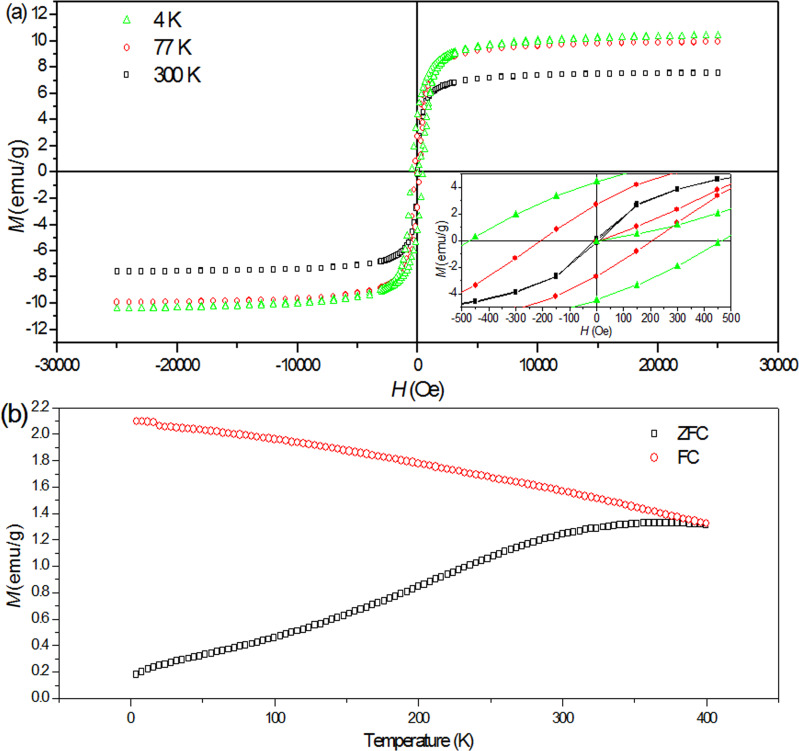
(a) Hysteresis loops of MnFe_2_O_4_/MWCNTs at 300, 77, and 4 K. (b) Zero-field and field-cooling magnetization curves of MnFe_2_O_4_/MWCNTs under an applied field of 50 Oe.

MnFe_2_O_4_/MWCNTs display ferrimagnetic behavior with very small hysteresis at room temperature (Figure 9). Hysteresis and magnetization increase with a decrease in temperature from 300 to 77 K and then to 4 K. The increase in magnetization with a decrease in temperature is a result of the decrease in thermal energy. The coercivity at 4 K is 471 Oe, which is significantly higher than the coercivity values reported for free MnFe_2_O_4_ nanoparticles [28]. Mössbauer results reveal that more Fe^3+^ is transferred from the octahedral sites to the tetrahedral sites in the partially encapsulated MnFe_2_O_4_ as compared to that in free MnFe_2_O_4_ nanoparticles. This leads to an enhancement of the magnetocrystalline anisotropy constant of partially encapsulated MnFe_2_O_4_, which in turn leads to the enhancement of coercivity [20]. Geng et al. [45] fabricated Fe-filled CNT arrays with high coercivity, which is primarily attributed to two factors: The first factor is the single-domain nature of the partially encapsulated Fe nanoparticles. Since the inner diameter of the CNTs is smaller than the single-domain size of the partially encapsulated Fe nanoparticles, the Fe-filled columns are magnetized by coherent rotation, which contributes to a large coercivity. The second factor is the large aspect ratio of the Fe-filled arrays characterized by a large shape anisotropy, which may lead to high coercivity.

The saturation magnetization of MnFe_2_O_4_/MWCNTs at room temperature is 7.6 emu/g, six times lower than the expected saturation magnetization value for free MnFe_2_O_4_ nanoparticles with a similar size [27]. This is attributed to the existence of the diamagnetic component arising from MWCNTs [46]. In order to investigate the blocking temperature, *T*_B_, of the MnFe_2_O_4_/MWCNTs, ZFC and FC measurements were carried out. The ZFC curve increased with temperature until it reached a maximum value, which corresponds to *T*_B_, at approx. 400 K, where the ZFC and FC curves overlap. The reported *T*_B_ for free MnFe_2_O_4_ nanoparticles of 9.3 nm in size is 397.7 K [47]. The estimated crystallite size of our partially encapsulated MnFe_2_O_4_ nanoparticles is approx. 10 nm. Therefore, 400 K is expected to be the blocking temperature for that particular size.

When comparing free and partially encapsulated MnFe_2_O_4_ nanoparticles, the blocking temperature is studied for both samples and observed to be approx. 400 K. The coercivity at 4 K for the partially encapsulated MnFe_2_O_4_ nanoparticles is 471 Oe, more than twice the value of 200.6 Oe reported for free MnFe_2_O_4_ nanoparticles. The saturation magnetization value at room temperature is 52 emu/g for free MnFe_2_O_4_, higher than the value obtained for partially encapsulated MnFe_2_O_4_/MWCNTs, which is 7.5 emu/g. This is due to the existence of a diamagnetic component in CNTs. In the literature, the reported value for MnFe_2_O_4_ nanoparticles of 12.5 nm in size is 55 emu/g [27].

From the above analysis it can be concluded that particle size has a major effect on the blocking temperature value. Shape, anisotropy, and single-domain features play a role in varying the coercivity value.

## Conclusion

In summary, free MnFe_2_O_4_ nanoparticles and MnFe_2_O_4_/MWCNTs were prepared using different methodologies. Free MnFe_2_O_4_ nanoparticles with sizes in the range of 5–24 nm were synthesized via an aqueous coprecipitation method. Saturation magnetization data at room temperature confirmed the existence of particles with an average size of 10–19 nm. XPS results supported by Mössbauer data validated the existence of Fe^3+^ and Mn^2+^ oxidation states in the MnFe_2_O_4_ nanoparticles. XPS data indicated the existence of traces of FeOOH and MnOOH at the surfaces of the nanoparticles. Although the blocking temperature is approx. 400 K, at 300 K the nanoparticles showed dominant ferrimagnetic and little superparamagnetic behavior due to the presence of small-sized particles. MnFe_2_O_4_ nanoparticles revealed a higher degree of inversion and a lower hyperfine field compared to the bulk. In agreement with the literature, particle size was observed to be the major parameter affecting the magnetic properties, such as saturation magnetization and blocking temperature. MnFe_2_O_4_ nanoparticles, attached to the external surface of the tubes and inside MWCNTs, were prepared using wet chemistry methods. XPS and Mössbauer data confirmed that Fe^3+^ and Mn^2+^ are oxidation states for partially encapsulated MnFe_2_O_4_ nanoparticles. Significant XRD peak shifts (i.e., a decrease in interplanar distances) were observed for partially encapsulated manganese ferrite nanoparticles compared to free MnFe_2_O_4_ nanoparticles. Upon attachment, evidence of electron transfer from MWCNTs to MnFe_2_O_4_ was observed. An increase in the hyperfine field of MnFe_2_O_4_/MWCNTs compared to free manganese ferrite nanoparticles was also detected. This can be assigned to two factors: strain from the MWCNTs walls and a higher inversion parameter. A large increase in coercivity of MnFe_2_O_4_/MWCNTs is ascribed to the single-domain feature and to a high aspect ratio. The blocking temperature was not affected by encapsulating manganese ferrite nanoparticles inside the multiwall carbon nanotubes.

## Supporting Information

File 1 Additional figures.
