# Supplementary material for: Free and partially encapsulated manganese ferrite nanoparticles in multiwall carbon nanotubes
Source: Beilstein J Nanotechnol. 2020 Dec 29;11:1891–904. doi: 10.3762/bjnano.11.170 (PMC7783029; doi:10.3762/bjnano.11.170)
Supplement: File 1 — Additional figures. [file Beilstein_J_Nanotechnol-11-1891-s001.pdf]

BEILSTEIN JOURNAL OF NANOTECHNOLOGY

Supporting Information

for

Free and partially encapsulated manganese ferrite nanoparticles in multiwall carbon nanotubes

Saja Al-Khabouri, Salim Al-Harthi, Toru Maekawa, Mohamed E. Elzain, Ashraf Al-Hinai, Ahmed D. Al-Rawas, Abbsher M. Gismelseed, Ali A. Yousif and Myo Tay Zar Myint

Beilstein J. Nanotechnol. **2020,** *11,* 1891–1904. doi:10.3762/bjnano.11.170

Additional figures

License and Terms: This is a supporting information file under the terms of the Creative Commons Attribution License (https://creativecommons.org/licenses/by/4.0). Please note that the reuse, redistribution and reproduction in particular requires that the author(s) and source are credited and that individual graphics may be subject to special legal provisions.
The license is subject to the *Beilstein Journal of Nanotechnology* terms and conditions: (https://www.beilstein-journals.org/bjnano/terms)

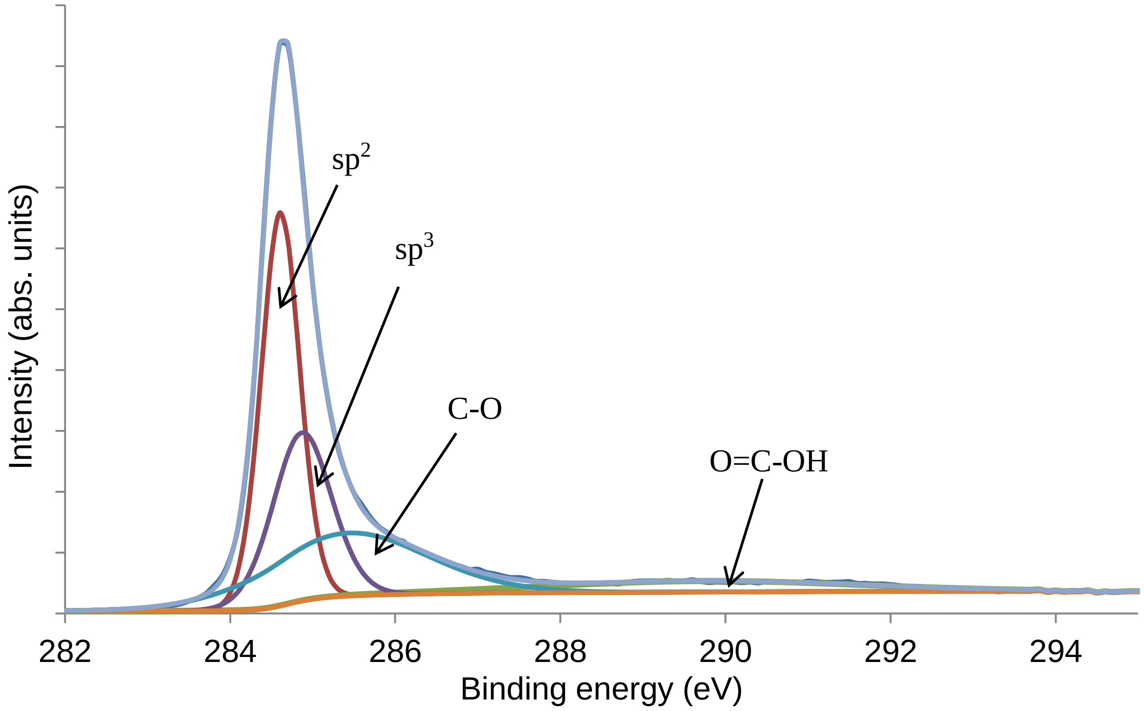

Figure S1: Core level XPS spectra of C 1s peak of MWCNTs.

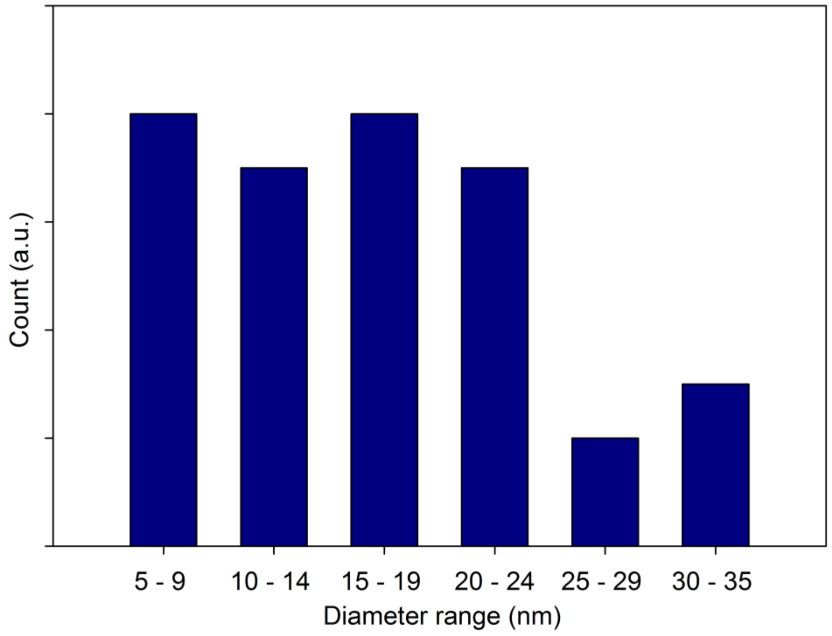

Figure S2: Particle size distribution of $MnFe_2O_4$ nanoparticles.
